# A thermoelectric materials database auto-generated from the scientific literature using ChemDataExtractor

**DOI:** 10.1038/s41597-022-01752-1

**Published:** 2022-10-22

**Authors:** Odysseas Sierepeklis, Jacqueline M. Cole

**Affiliations:** 1 Cavendish Laboratory, University of Cambridge, J. J. Thomson Avenue, Cambridge, CB3 0HE UK; 2 ISIS Neutron and Muon Source, STFC Rutherford Appleton Laboratory, Harwell Science and Innovation Campus, Didcot, Oxfordshire OX11 0QX UK

**Keywords:** Thermoelectrics, Cheminformatics, Energy, Chemical physics

## Abstract

An auto-generated thermoelectric-materials database is presented, containing 22,805 data records, automatically generated from the scientific literature, spanning 10,641 unique extracted chemical names. Each record contains a chemical entity and one of the seminal thermoelectric properties: thermoelectric figure of merit, *ZT*; thermal conductivity, *κ*; Seebeck coefficient, *S*; electrical conductivity, *σ*; power factor, *PF*; each linked to their corresponding recorded temperature, *T*. The database was auto-generated using the automatic sentence-parsing capabilities of the chemistry-aware, natural language processing toolkit, ChemDataExtractor 2.0, adapted for application in the thermoelectric-materials domain, following a rule-based sentence-simplification step. Data were mined from the text of 60,843 scientific papers that were sourced from three scientific publishers: Elsevier, the Royal Society of Chemistry, and Springer. To the best of our knowledge, this is the first automatically-generated database of thermoelectric materials and their properties from existing literature. The database was evaluated to have a precision of 82.25% and has been made publicly available to facilitate the application of data science in the thermoelectric-materials domain, for analysis, design, and prediction.

## Abstract (summary)

**Table:** 

Measurement(s)	thermoelectric figure of merit, Seebeck coefficient, thermal conductivity, electrical conductivity, power factor
Technology Type(s)	ChemDataExtractor

## Background & Summary

Thermoelectric materials can convert heat into electricity, and vice versa. With the increase in global energy demands and the adverse impact which current methods of electrical generation have on the environment, thermoelectric materials present themselves as an opportunity towards a more sustainable future. Most electrical and mechanical processes generate vast amounts of waste heat which could be harvested through the application of thermoelectric devices. Such devices are lightweight, scalable, solid-state, and with large and reliable working lifespans^1^.

The effectiveness of a thermoelectric material at converting heat into electricity, and vice versa, is dictated by the thermoelectric figure of merit, *ZT* ^2^. *ZT* is defined by Eq. 1, where *S* is the Seebeck coefficient, *σ* is the electrical conductivity, *κ* is the thermal conductivity, and *T* is the temperature of the material.

1 $$ZT=\frac{{S}^{2}\sigma }{\kappa }T$$

To achieve a high thermoelectric figure of merit, a material should combine a high Seebeck coefficient and/or electrical conductivity, coupled with low thermal conductivity. The interdependence between these quantities makes this a challenging endeavour^3^ and several methods are being employed, such as doping and nanostructuring, to increase the thermoelectric performance of materials. The field attracts significant interest, with an increasing number of articles being published in recent years.

Traditionally, the discovery of novel materials was guided by the intuition of researchers and trial-and-error process. However, recent advances in the field of data science and the development of off-the-shelf machine-learning algorithms offer the potential to streamline materials discovery^4^. As the name suggests, for data-driven methods to be effective, a large dataset of relevant information is required.

In the field of thermoelectrics, Gaultois *et al*. first performed a data-driven review of thermoelectric materials based on a manual endeavour, which catalogued the five aforementioned thermoelectric quantities, for different temperatures, and different families of thermoelectric materials^5^. Information extracted from 100 articles resulted in a database with 1,100 data records, essentially pioneering data-driven analysis in the thermoelectric-materials domain. In a subsequent study^6^, these data were combined with *ab-initio* calculated electronic-structure properties from Hautier *et al*.^7^ and Pymatgen^8^, to train a machine-learning system, which operates as an online recommendation engine (http://thermoelectrics.citrination.com). 25,000 computationally-generated compounds were screened, and a novel compound, “far” from the chemical space of familiar thermoelectric compounds, was suggested as a promising, yet unstudied, thermoelectric material.

*Ab-initio* Density Functional Theory (DFT) calculations are a typical source of data for thermoelectric applications^9^, allowing the computation of values for different thermoelectric properties spanning thousands of materials. Gorai *et al*. have developed the Thermoelectric Design Lab^10^, an online tool which ranks thousands of compounds based on their calculated thermoelectric properties, using a bespoke predictor^11^, which has been shown to predict *Z*, at peak *ZT*, within a factor of approximately two. According to the website, *“the database contains calculated transport properties and thermoelectric performance rankings of 2701 materials”*. More recently, Tshitoyan *et al*.^12^ trained word embeddings on materials-science abstracts to discover compounds which are likely to belong to the field of thermoelectric materials, but had not yet been studied as viable candidates *per se*.

In such studies, authors regularly comment on the potential that a large and reliable thermoelectric-materials database can provide. While *ab-initio* computationally-generated databases have the ability to produce massive quantities of data, they suffer from the need to apply approximations which challenge their reliability. Contrastingly, manually-curated databases can be highly precise, but require significant time investment to build by experts in the field. The use of text mining to automatically extract data from the scientific literature can combine the accuracy of experimental data, with the high-throughput capacity offered by computational means. This paper showcases how the chemistry-aware natural language processing toolkit, ChemDataExtractor 2.0, based on software developments by Cole and co-workers^13,14^, was used to generate a thermoelectric-materials database, through automatically extracting data from a large corpus of relevant research papers.

To the best of our knowledge, this is the first auto-generated thermoelectric-materials database from the scientific literature. Adaptations were made to ChemDataExtractor, relevant to the thermoelectric-materials domain, which may possibly be extended to other material properties as well. The workflow of the automatic generation of our database includes article retrieval, pre-processing and data extraction, data cleaning, post-processing, and evaluation. The resulting database presents the potential to facilitate thermoelectric-materials discovery through materials informatics, as well as function as a user look-up table to inform scientists on certain materials and their properties.

## Methods

### Article retrieval

The first step in the database-generation workflow was to employ web scraping in order to extract a large number of articles found online, from scientific publishers. Specifically, 40,488 articles from Elsevier, 4,983 articles from the Royal Society of Chemistry (RSC), and 15,392 articles from Springer were scraped using the keyword ‘thermoelectric’ for searching. This resulted in a total of 60,843 articles from which thermoelectric information was extracted. There were 1,904, 603, and 2,440 open-access articles in these sets, from Elsevier, the RSC, and Springer, respectively. ChemDataExtractor provides python code for web scraping, making use of the ‘urlib3’ and ‘requests’ HTTP client python libraries, which was used to download the articles onto a local machine. Using these scrapers, the Elsevier articles were obtained in Extensible Markup Language (XML) form and the RSC articles in Hypertext Markup Language (HTML) form. The Springer articles were downloaded in simple text form, using a separate scraper. Elsevier and Springer provide a representational state transfer (REST) service, in the form of an application programming interface (API), which supports online requests; a valid API key is required, which is distributed by the publishers. Different search criteria can be specified, such as query words, date of publication, etc., which allows the retrieval of the desired Digital Object Identifiers (DOIs) and articles. The RSC had not yet established an API during the timeframe of this work; therefore, documents from the RSC were scraped using the Mozilla Firefox Gecko WebDriver, which provides the API to communicate with the browser and download the articles.

### Document processing

ChemDataExtractor takes in the downloaded documents and uses bespoke readers to process their information. XML and HTML documents have a hierarchical structure, with semantic markup tags, such as a <head> tag which contains information regarding the DOI, authors, and title of a document. ChemDataExtractor uses these tags to identify the significant features of each document such as the titles, headings, the abstract, and figures. The text of the document itself is then collated, to create a continuous and uniform stream of text elements. The stream is stored in the Document object of the ChemDataExtractor toolkit and follows a predefined order, which allows processing each document in the same manner, irrespective of the original setup, forming the basis for further processing. Text files downloaded from Springer are processed ‘as is’ since they lack semantic markups.

### Natural language processing

ChemDataExtractor employs state-of-the-art Natural Language Processing (NLP) techniques which have been tailored to the chemistry and physics domain, such as bespoke Sentence Splitting, Tokenisation, Part-of-Speech (POS) Tagging, and Chemical-Named Entity Recognition (CNER), to facilitate data extraction. The NLP code of ChemDataExtractor remained mainly unchanged for the purposes of this work, but targeted adaptations were performed to improve CNER for the thermoelectric-materials domain. For example, since many thermoelectric compounds contain dopants, CNER rules were extended to extract chemical entities in several other formats which express doping, whether qualitatively or quantitatively. Table 1 presents different doping forms and examples of chemical entity mentions (CEMs) which our adaptation of ChemDataExtractor can identify and extract. Furthermore, prefixes and suffixes relevant to the thermoelectric domain were added, such as ‘pure’, ‘undoped’, ‘mono/polycrystalline’, ‘superlattice’, ‘p/n-type’, and several others. These affixes can capture information about the structuring, dimensionality, crystallinity, semiconductor type, or doping of a material. The adapted thermoelectric-centred version of ChemDataExtractor is available at https://github.com/odysie/thermoelectricsdb/tree/main/chemdataextractor_thermoelectrics.

**Table 1:** Different types and examples of CEMs which can be extracted, following adaptation of the default ChemDataExtractor toolkit for the thermoelectric-materials domain.

Type	Examples
Simple	SnSe, PbTe, bismuth telluride, Na_0.03_Sn_0.97_Se_0.7_S_0.3_
CEMs with prefixes and suffixes	p-type SnSe crystals, polycrystalline Na_0.03_Sn_0.97_Se, Bi_0.4_Sb_1.6_Te_3_ bulk,
	2D layered In_4_Pb_x_Sn_y_Se_3_
Composites and other combinations	PEDOT-BiTe3 nanorod composites, Bi-Sb matrix, Bi_2_Te_3_/Sb_2_Te_3_ superlattice
Simple doping	Cr-doped PbSe, iodine-doped SnSe, Ba-filled Ba_0.25_Co_4_Sb_12_, Sb-doped n-type Ti_0.5_Zr_0.25_Hf_0.25_NiSn_0.998_Sb_0.002_
More advanced, explicit doping with percentages	Sn_0.98_Bi_0.02_Te−1%HgInTe_2_, PbTe + 2% Na + 4% SrTe, PbT-4% SrTe doped with 2% Na, polycrystalline hole-doped SnSe alloyed with 5% lead selenide, 10 wt % Al-doped ZnO thin film, PbT-SrTe (4 mol %) doped with 2 mol % Na
Co-doping	germanium telluride co-doped with BiTe and Cu

### Information extraction

ChemDataExtractor 2.0 offers three distinct methods of information extraction from text, which can function independently or complementary to each other. The first one involves the explicit crafting of statements that utilise regular expression (regex) patterns and POS tags to extract information through a rule-based approach. The second method is automatic parsing, which relies on objects of dimensions, units, and models built in ChemDataExtractor to define a property for extraction, similar to the way it would be defined using SI base units, having a measurable quantity with a numerical value and possible units. ChemDataExtractor also includes template parsers, which come pre-packed with hand-crafted rules for information extraction and can be used off-the-shelf or as a basis to develop new rules depending on the targeted field. The third method deploys a modified version of the Snowball algorithm^15,16^ which can be trained in a semi-supervised manner on a corpus of documents and then used to probabilistically extract information at the sentence level of documents. Methods one and three have already been used to generate material databases on their own^17^, or synergistically^16^. In order to examine its advantages and disadvantages, as well as gauge its applicability and potential, automatic parsing was adapted and used in this work.

The built-in framework of ChemDataExtractor was used to define the necessary models, units, and dimensions for the extraction of the thermoelectric figure of merit, electrical conductivity and resistivity, thermal conductivity (total, electronic, and lattice contributions), Seebeck coefficient, and also the power factor. The power factor is a compound quantity used to describe thermoelectric materials, typically denoted as *PF*, and is equal to *S*^2^*σ*. Each of these five models nests a temperature model, meaning that every extraction also requires the retrieval of an associated temperature, given the temperature-dependent nature of these properties. The temperature model inherits from the temperature quantity object, which is available through the default installation of ChemDataExtractor 2.0. The temperature model extracts numerical values and different possible units, such as Kelvin, Celsius, or Fahrenheit. However, in scientific articles, the value of a thermoelectric quantity is sometimes followed by a string of words, such as *“at room temperature”*, to denote that the reading was taken near 295 Kelvin. Therefore, a new field was introduced in the models, by leveraging the StringModel functionality of ChemDataExtractor. This field allows the extraction of specific text patterns, in order to capture room-temperature mentions, which are translated into a numerical value post-extraction.

Material-science articles include complex information about the processing, structure, properties, and performance of a material. Articles about thermoelectric materials are complex due to the frequent use of doping, nesting of properties to temperature readings, and synthetic processes involved at specific temperatures which affect the values of measured properties, potentially causing many false-positive extractions. Therefore, the scope of textual extraction was limited to one sentence at a time, using the automatic sentence-extraction capabilities of ChemDataExtractor 2.0 through the provided AutoSentenceParser (ASP). As discussed, the ASP does not rely on bespoke rules but takes in the defined property model and uses the contained fields to extract a single record from sentences using a first-come-first-served approach. Any fields that are found in the text are extracted and linked together, connecting CEM, property specifier, units, values, etc. into a single data record. While the linking of the fields follows the first-come-first-served approach, the fields themselves have restrictions imposed on them through a hybrid of machine-learning and hand-crafted rules, which attempt to extract the correct information. Naturally, different rules pertain to different fields, such as CEMs, numerical values, units, and other descriptors which have been adapted within ChemDataExtractor for application in the thermoelectric-materials domain. This limits the extracting capabilities of ASP to one record per sentence, as well as sometimes falling victim to specific structures of sentences which include more than one set of materials or properties. The issue is exacerbated due to the dimensionless nature of the thermoelectric figure of merit, *ZT*, which does not restrict its numerical value extractions through the expectation of the existence of units, unlike the other properties extracted in this work. Furthermore, since researchers in the thermoelectric-materials domain typically have the principal goal of increasing the thermoelectric figure of merit, *ZT*, its data extraction hosts frequent use of comparative sentence structures, where novel records are compared to materials of similar types and families, or against high-performing standards. The prevalence of such sentences poses an additional hurdle for the default ASP capabilities of ChemDataExtractor 2.0, which may not be as prevalent in other fields with less frequent use of direct comparisons.

**Fig. 1:**
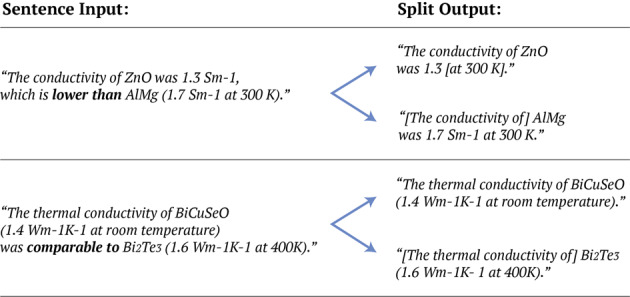
Example illustration of the splitting and stitching step, where the bold letters indicate the trigger words of each sentence and the square brackets indicate information transferred from a different part of the split sentence.

In order to ameliorate these issues, an intra-sentence ‘splitting and stitching’ step was employed prior to information extraction, to simplify sentences and therefore reduce the number of false-positive records, while also increasing the number of the true-positive ones. Specifically, if a nested model is provisionally identified in a sentence, and the sentence contains trigger words which indicate the comparison of multiple records, then the sentence is split based on the trigger word(s). Each part of the sentence is then combined with the identified specifier and temperature from the provisional record, only if no such information exists in the split. Following the examples seen in Fig. 1, a specifier or temperature will only be transferred in the resulting parts of the sentence if that information cannot be found therein. In the first example, the temperature reading and specifier are transferred to the first and second part of the sentence, respectively, as indicated by the square brackets. In the second example, only the specifier is transferred to the second part of the sentence, which lacks a specifier, while information about temperature is not transferred at all, since it can be found in both split parts of the sentence.

This process may sometimes introduce unwanted artefacts in the split sentences, leading to incorrect data extractions. However, the majority of changes are beneficial, leading to an overall decrease of false positives and an increase of true positive records.

Furthermore, a range parameter was included in the ASP, which sets boundary conditions on the acceptable values during data extraction. This was employed during the extraction of *ZT*, using a range within the extremes of known records for thermoelectric materials. At the time of writing this paper, single crystal *SnSe* held the record for *ZT* at 2.6^18^, amongst well-studied materials. Therefore, the range was set to be 0 to 3 for viable data extractions of *ZT*. Another pre-parsing step was added which masks any temperatures pertaining to fabrication processes (such as sintering at specific temperatures), in order to avoid false positive data extractions between the temperature of a material during property measurements and the temperature of the process through which a material was created. Finally, more fields were added which extract information about the processing and treatment conditions which were involved in the creation of a material, such as annealing, sintering, milling, and others, at possible pressures or the masked temperatures described earlier. Chemical names that consist of single elements were excluded from viable data extractions, since thermoelectric materials are typically compounds of at least two chemical elements. Single-element doping was naturally accepted, with an additional field added to extract doping information which may be included in a sentence, but is not part of the chemical entity; e.g. “*BiCuSeO showed a ZT of 1.3, following Cl doping”*. The Data Records section describes the fields which can be included during the extraction of a data record, as well as a brief explanation of the information that they hold.

Following the definition of functioning property models as well as the pre-processing steps described above, ChemDataExtractor allowed for the automatic extraction of information from the selected corpus of articles. A supercomputer was employed for the data extraction process, owing to the large amount of approximately 60,000 articles that comprised the data source, and the moderate computational demands that were imposed by the adapted processing pipeline of ChemDataExtractor. Thereby, the supercomputer *Cooley*, of the Argonne Leadership Computing Facility (ALCF)^19^, Illinois, U.S.A., was used. Scripts to be run were submitted as jobs, which requested a certain number of nodes from the supercomputer, wherein the system queues the jobs and allocates computing resources accordingly. Scripts were written in python and employ the mpi4py module^20^, which follows the message passing interface (MPI) protocol for parallel programming. Different scripts were coded for subsets of 10,000 articles for each model using 10–20 nodes each, and these ran for about 10 hours. The five aforementioned thermoelectric properties were the primary targets for data extraction; although models for six properties were used during data extraction, the additional one being electrical resistivity, which is then translated to conductivity using their inverse relationship.

### Data cleaning, normalisation, and inference

Following the data extraction process, code was written to clean the resulting databases, by filtering out data records with exorbitant entries, such as compounds without any alphabetical characters, or with names of more than 200 characters long. More specific cleaning steps were performed, such as filtering out chemical names with common problematic forms, e.g. a numerical percentage followed by a chemical entity, which most likely refers to doping, rather than the compound itself. These steps can be found commented in the code uploaded onto https://github.com/odysie/thermoelectricsdb. Each data record was tagged with the publisher (Elsevier, the RSC, or Springer) of the source article, whether the article is open-access or not, and its year of publication. Electrical resistivity values were normalised to electrical conductivity values following their inverse relationship. Furthermore, the room-temperature mentions were translated into numerical values, and all the values of the extracted quantities were normalised to a single unit for each quantity. Where both temperature values and room-temperature mentions had been extracted, precedence was given to the room-temperature mention. This is because room-temperature mentions are more likely to refer to the temperature of the material during measurement, while numerical temperature values may refer to other instances, such as chemical or mechanical processes. Thermal conductivity was distinguished between its electron, phonon, and total contribution, and electrical conductivity was classified as normal or ionic, according to regex rules that were run on the extracted specifier. Furthermore, the interdependence between extracted properties allowed the inference of new data records, which will be described in the Technical Validation section.

## Data Records

The full database is publicly available for download from *Figshare*^21^, in three standard formats: CSV, JSON, and MongoDB. This section describes the records found in our database. The field Specifier contains the textual information that is required for each property to be extracted. This Specifier field may also contain information which indicates that the value of a property lies at its extreme ends, or is an average, since optional prefixes such as ‘peak’, ‘max/min’, ‘highest/lowest’, ‘mean/average’ etc. are also extracted if they are found to precede the specifier. Information about the semiconducting type of the compound can also be found as part of the specifier. The field Model distinguishes between one of the five extracted properties: the thermoelectric figure of merit, thermal conductivity, Seebeck coefficient, electrical conductivity, or power factor. In the case of thermal conductivity, the field Model_Type further differentiates between the possibilities of ‘lattice’, ‘electronic’, and ‘total’ thermal conductivity, while in the case of electrical conductivity it distinguishes between ‘electronic’ and ‘ionic’ conductivity. The field Name is the name of the extracted chemical entity. The Label field refers to the value of a variable composition label that is possibly found in the compound. Value and Units are the values and units of the related property, respectively, normalised according to each model to support uniformity. The Units section is not populated for the dimensionless case of *ZT*. Each data record has an associated Temperature_Value and Temperature_Units field, normalised to Kelvin. Typically, the Value and Temperature_Value fields are a list with a single numerical value. When the extraction represents a range, there are two components, with the first and second representing the minimum and maximum value of the range, respectively. There are also averages of these values, found in Temperature_Average and Value_Average columns. The field Editing contains components or procedures which describe the material, such as doping, but were not extracted as part of the chemical name. The field Pressure is populated with a list, where the first component is the extracted numerical value and the second component is the units of the pressure under which the sample was studied. The field Process contains information about the extracted process of the data record, while the field Direction_of_Measurement offers information about the axial or planar direction of the property measurement. The field Resolution attempts to resolve simple combinations of CEMs into ‘host’ and ‘dopant’ components, while distinguishing between different methods of combination, such as ‘doping’, ‘addition’, ‘mixture’ etc., and identifying the amount involved, if specified. The field contains a python dictionary with this information, where this resolution of components has been performed post data extraction. For example, the compound name ‘10 at. % *Nb* doped *TiO*_2_’ would be resolved to {‘host’: ‘*TiO*_2_’, ‘dopant’: ‘*Nb*’, ‘amount’: ‘10 at. %’, ‘method’: ‘doping’}. This procedure has not been evaluated on its efficacy and mainly serves as a recommended direction towards data-science applications. Finally, the fields DOI, Title, Access_Type, Publisher, Journal, Authors, and Publication_Year contain the relevant information about the article from which the data record has been extracted.

## Technical Validation

The metrics of precision and recall were used to evaluate the quality of the database. Precision is the percentage of correct data extractions relative to the total number of extractions, and recall is the percentage of correct data extractions with respect to the true number of records that are present in the articles, as described by Eqs. 2 and 3.

2 $$Precision=\frac{TP}{TP+FP}$$

3 $$Recall=\frac{TP}{TP+FN}$$

where TP, FP, and FN are the number of true positive, false positive, and false negative records, respectively. A record was considered to be a TP if the Name, Editing, Model, Value, Units, and associated Temperature_Value and Temperature_Units fields were found to match the sentence of the document from which they were extracted; and a FP if any of these data were incorrect. A FN is a record which exists in an article, about which no data extractions have been made by our system, i.e. a *missed* record.

**Fig. 2:**
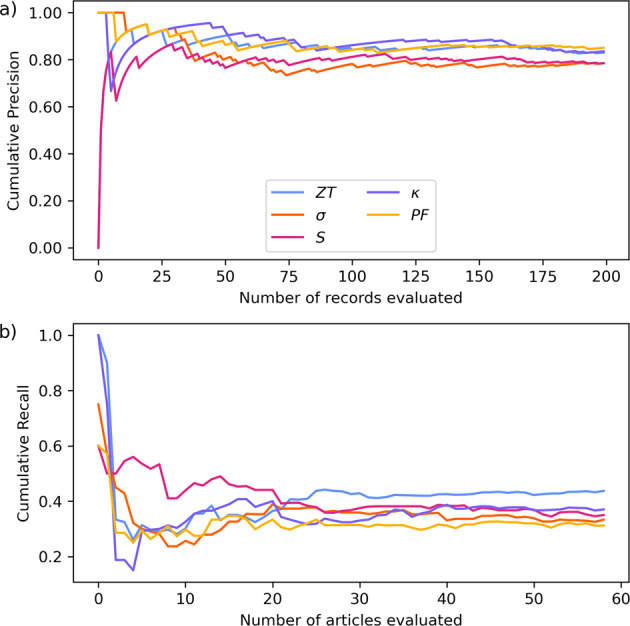
Cumulative average (**a**) precision and (**b**) recall for the five extracted thermoelectric properties, showing convergence of values, as a function of successively evaluated data records or articles.

In order to evaluate sentence precision, 200 records of each of the five properties were randomly sampled from the database, prior to the inference procedure (*vide infra*), across a total of 1000 records. The data records were sampled from open-access articles where possible, and supplemented with records from subscription-type articles where necessary. The number of 200 records was chosen based on tests which indicated that this many records will suffice to demonstrate a convergence in precision. As shown in Fig. 2a), the precision score flattens out after about half of these records have been used in the evaluation, suggesting that the metric is representative of the entire database. In order to evaluate recall, 60 articles which yielded data records from at least 3 different models were randomly sampled from the database. Similar to precision, convergence is observed over this span of sampling, as seen in Fig. 2b). Of these articles, 53 were open-access, supplemented by subscription-type articles, in order to fairly represent each publisher. The shape of these graphs are, naturally, dependent on the order by which the articles are selected (or presented) for evaluation, but shuffling the order was found to produce similar convergence trends.

Table 2 showcases the results for the precision, recall, and number of records for each property. The weighted average sentence precision of all properties is 82.25%. This weighting is according to the fraction between the number of data records for each model and the total number of data records for all models, to better indicate the precision of the entire database. Precisions of individual properties range from 78.5%–85%. The moderate variation of this range (6.5%) suggests good reliability and consistency within data extractions. The weighted average recall of the five models is 39.23%. The low recall is in part due to the restricted scope of data extraction, being at the sentence level, while several records span more than one sentence (typically two, and occasionally five or more). The discrepancy between the high recall of *ZT* and the other four models with dimensions is attributed to units extraction. In some cases, the units are expressed in an unconventional (or even, strictly speaking, *incorrect*) way, which ChemDataExtractor does not account for, leading to a higher ratio of false negatives for the models with dimensions. This is particularly true for the power factor, which involves a comparably complicated data extraction, given that it manifests different exponents, both in its units (*Wm*^−1^*K*^−2^), and, on occasion, even in its specifier (*S*^2^*σ*, $\sigma {a}^{2},\frac{{S}^{2}}{\rho },$ etc.). The authors consider the most important metric to be the estimated precision of the overall database, which was found to be as high as 82.25%. This priority reflects the tenet that it is better to have a smaller database that contains a high proportion of correct data records than a larger database whose records are largely erroneous.

**Table 2:** Precision, recall, and number of records for each model, nested with temperature.

Model	Precision (%)	Recall (%)	Number of records
*ZT*	83.00	43.71	11668
Thermal conductivity	83.50	37.00	3701
Seebeck coefficient	78.50	33.00	2584
Electrical conductivity	78.50	34.97	2728
Power factor	85.0	31.18	2224
Weighted Average	82.25	39.23	N.A.

Typically, high precision is achieved, through sacrificing recall. The large amounts of chemical and numerical information that are found in materials-science articles, coupled with the desire to extract each property at the correctly associated temperature using the automatic parsing capabilities of ChemDataExtractor, led to the restriction of extracting data at the sentence level. Nevertheless, the coding adaptations performed in ChemDataExtractor to serve this material domain allowed for the creation of a relatively sizable and precise database of thermoelectric properties; meanwhile the developed methods may be extendable to property models in other material domains, especially the intra-sentence splitting-and-stitching procedure, the value range restriction parameter, and the extended CNER for doped compounds.

The precision of this database is comparable to other NLP approaches in materials science. Court & Cole extracted Curie & Néel temperatures across 39,822 data records and achieved a precision of 73% using a rule-based approach and the introduction of a modified Snowball algorithm^16^. Huang & Cole used a rule-based approach to achieve an 80% precision for a database of battery materials that comprises of 292,313 data records^17^. Our approach is similarly automatic and expected to be applicable to any other standalone property, with or without dimensions, which nests or does not nest temperature. Similarly, it is expected to be extendable to any property, which nests any other property. The database of battery materials^17^ offers human-like precision for 292,313 unique records, extracted from 229,061 articles, achieving a much higher number of records than ours, with similar precision. This demonstrates both the power of high recall (80% compared to our 39%), as well as that of being able to source many articles from a large corpus of existing literature. For comparison, when Springer returned 28,396 search results for the word ‘thermoelectric’, it returned 269,351 results for the query word ‘battery’, which is about an order of magnitude higher. Finally, it is noted that the methods of data extraction are different between the database of Huang & Cole and ours (rule-based versus modified automatic extraction), and while the number of extracted properties is the same, all of our records are nested with an additional property: temperature. Databases with fewer records or lower precision than ours have already been demonstrated to suffice for data-driven materials discovery^22^. However, the additional dimension introduced by the temperature dependence in this study may warrant the *a priori* data extraction of a higher number of records to perform such a task, thereby calling for improved recall during the data extraction stage or a larger corpus from which to extract relevant data.

### Aggregated data

As discussed, our extracted database of thermoelectric materials contains 22,805 records. This contains 10,641 unique extracted chemical names, although these include names which may refer to the same compound but are expressed differently (e.g. 5% *Cl*-*doped ZnO* and *Zn*_0.95_*O*_0.9_*Cl*_05_), leading to an overestimation of the true value. In order to aggregate the extracted information, the normalised model values of the database were collected according to matching compounds (and their paraphernalia, such as labels, pressure etc.), and the DOIs from which each record was extracted. The resulting database records feature unique compound, temperature, and DOI entries, with any number of the five possible properties at that temperature. Wherever there were multiple values for the same set of the fields described, an average of the value was taken. This procedure has brought the number of data records of the resulting database down to 18,509, which contain 19,311 different property values. In this database, the Seebeck coefficient has been squared, only the total thermal conductivity component has been kept, and no distinction is made towards ionic electrical conductivity. This representation facilitates comparisons between existing records, or the inference of new records, due to the intrinsic relationships that exist between the thermoelectric properties described in Eq. 1.

Thereby, comparisons among the extracted records were performed, wherever possible, using the equations *PF* = *S*^2^*σ* and *ZT* = *PF/Tκ*. When all of the terms involved in one of these equations were included in a data record, then a target value could be compared, by making it the subject of the equation at hand and substituting the dependent variables. The rest of the values were used to calculate the target value, and that result was contrasted with the extracted target, taking an absolute percentage difference. The process was then repeated, taking a different quantity as the one to be calculated. Equation 1 itself was omitted from the inference procedure, owing to the high probability of error and low number of eligible data records, which stems from the necessary presence of five different extractions per record. There are only two ways of performing these comparisons, on symmetry grounds, which can be seen as comparing the left-hand side, LHS, to the right-hand side, RHS, of the equations, as seen in Eq. 4.

4 $${\rm{Absolute}}\; \% \;{\rm{difference}}=\frac{| {{\rm{LHS}}}_{{\rm{calculated}}}-{{\rm{RHS}}}_{{\rm{extracted}}}| }{{{\rm{RHS}}}_{{\rm{calculated}}}}$$

**Fig. 3:**
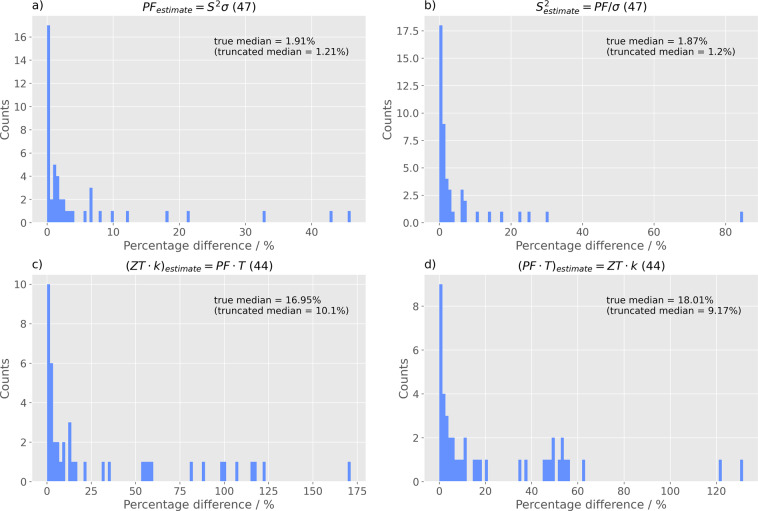
Histograms of the absolute percentage differences (up to 200%) where different quantities were selected as the subject of the equation: (**a**) *PF* = *S*^2^*σ*, (**b**) *S*^2^ = *PF*/*σ*, (**c**) *ZT*·*κ* = *PF*·*T*, and (**d**) *PF*·*T* = *ZT*·*κ*. The bracketed numbers in the titles of a histogram refer to the number of aggregated records involved in the comparison.

The four comparisons resulted in low medians for the absolute differences. Although this does not imply a good correctness of our data extractions, it serves to showcase that there is consistency between these extractions from each article. Figure 3 shows the histograms of these absolute differences, truncated after 200%, for presentability. Regardless, the true median prior to truncation is displayed. As expected, the comparisons stemming form the second pair of comparisons show a higher median in the absolute difference, since the involvement of four extractions compared to three increases the probability of disagreement between the quantities. A Comparison field has been added to the aggregate database, which holds a python dictionary with the percentage differences calculated per data record, where any are appropriate.

**Fig. 4:**
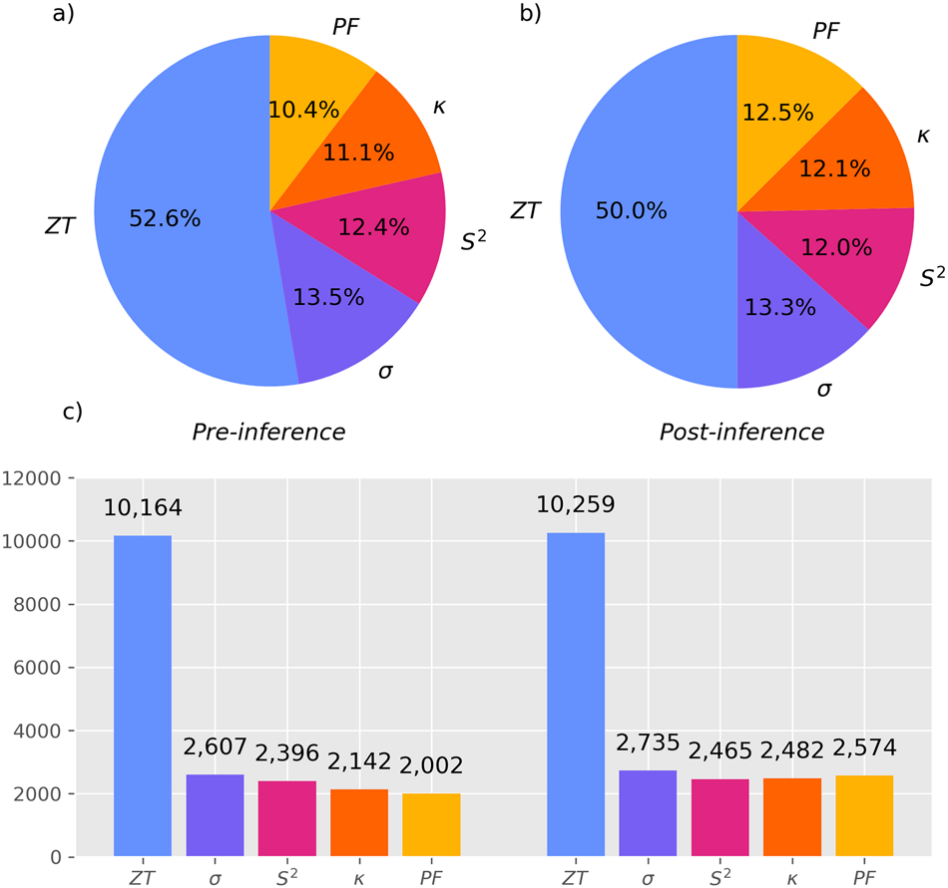
Pie charts (**a**) and (**b**) comparing the percentage of total records for each model, before and after inference, respectively. Bar chart (**c**) presents the exact number of records for each model, before and after inference.

The aggregate database also allows for the inference of new values, based on these thermoelectric equations and Eq. 1, whenever a sufficient number of property values where found in a data record. The process of inference has generated an additional 1,204 unique records. Figure 4 demonstrates how many data records have a given number of models associated with them, as well as their prevalence in the database, shown as a ratio, before and after inference.

The vast majority of the extracted records are thermoelectric figures of merit, as expected, since this is the most sought-after quantity in the thermoelectric-materials domain, while it also affords more extractions due to its dimensionless nature. Therefore, the property with the lowest ratio of newly-inferred records is the thermoelectric figure of merit. Depending on the properties involved in each inference, the evaluated precisions of those constituents can be used to estimate the precision of the inferred property, as can be seen in the inference_estimated_precisions.csv file uploaded onto https://github.com/odysie/thermoelectricsdb/tree/main/supplementary_material.

All data records in the aggregate database have at least one property, by design; but, prior to inference, only 1.34% of them contained three or more properties. The inference step increases that percentage to 6.21%. Naturally, the number of data records with exactly one property did not change. The number of data records with exactly 2 properties decreased, while the number of records with exactly 3 properties increased, as inferences were performed where possible. The number of data records with exactly 4 properties vanished, as all those occurrences were transformed into records with all 5 properties. The aggregated database can be downloaded from *Figshare*^21^. The fields of the database are similar to the ones found in the database prior to aggregation, as described in the Data Records section, with the main difference that there are five distinct fields for each of the possible property values for each data record, rather than one property per record. These fields are ZT, PF, S^2, s, and k for the thermoelectric figure of merit, power factor, Seebeck coefficient (squared), electrical conductivity, and thermal conductivity, respectively. There are two additional fields, Original_Counts and New_Counts which inform on how many of these five thermoelectric properties were included in each data record, prior to and after inference, respectively. Finally, there is an additional Inference field which shows which, if any, properties participated in the inference procedure.

**Fig. 5:**
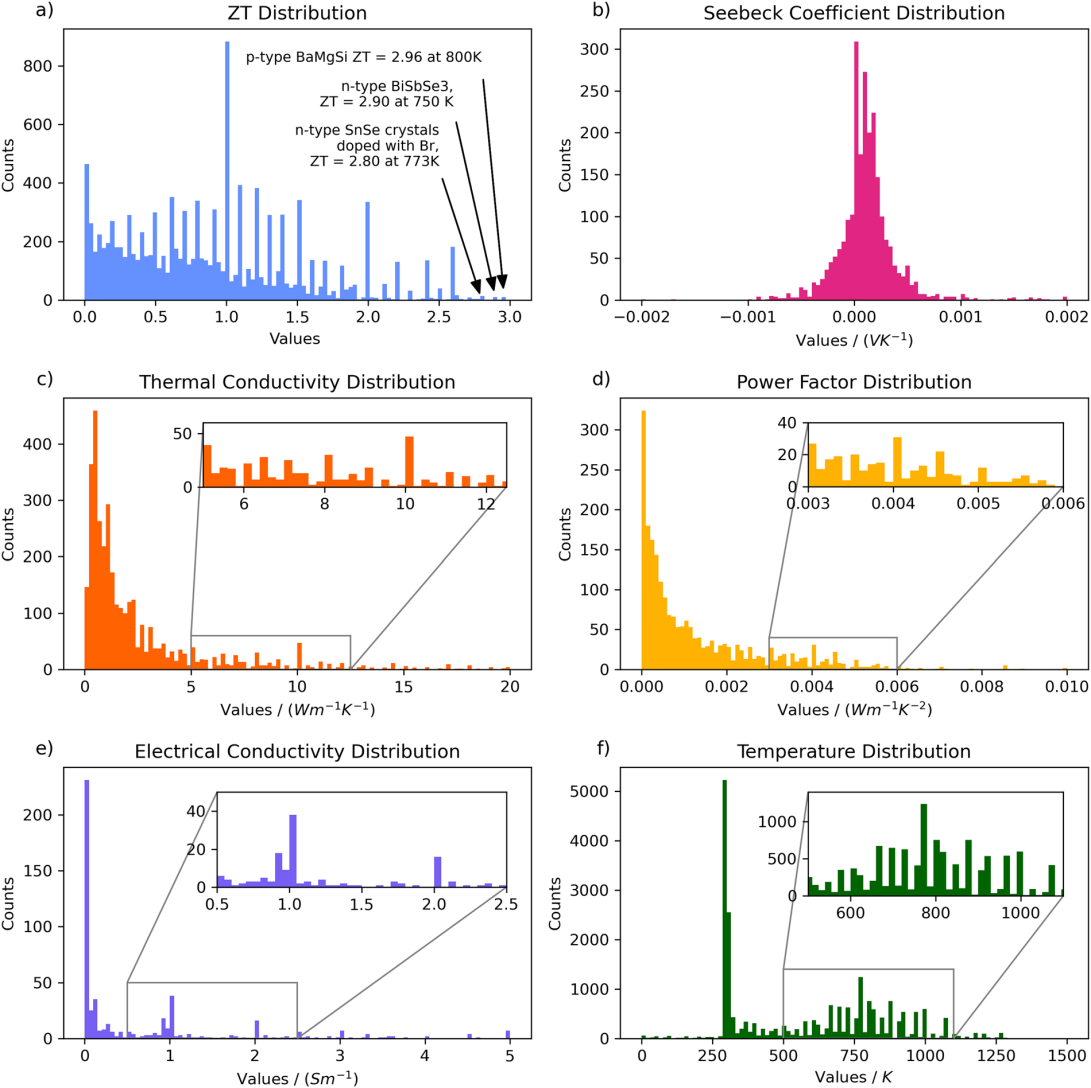
Data distributions of the five properties: (**a**) *ZT*, (**b**) thermal conductivity, (**c**) Seebeck coefficient, (**d**) power factor, (**e**) electrical conductivity, and (**f**) temperatures (across all models).

Figure 5 presents the histograms of the five extracted properties. Some artefacts appear in the distributions. In particular, for the case of *ZT*, spikes can be seen near integral decimal values, which is considered to reflect the tendency of researchers to round numbers to meet integral digits, as well as quoting whole numbers as benchmarks which have been, or which should be, met. The significant spike at *ZT* = 1, (Fig. 5a), can be attributed to the use of unity as a benchmark value for many thermoelectric materials. We can also identify a significant spike near room temperature at the temperature distribution, (Fig. 5f), which can be attributed to the high emphasis placed on room-temperature-operating thermoelectrics, as well as a secondary family of spikes near 800 K. Studying the temperature distributions individually for each model reveals that this family of peaks stems primarily from the *ZT* model, which may indicate that a lot of thermoelectrics have their highest values around 800 K, hence are being quoted more often at those temperatures; however, further analysis which is beyond the scope of this data descriptor paper is required. As pointed out in the *ZT* distribution (Fig. 5a), the best-performing material in terms of *ZT* found in our database is p-type *BaMgSi*, which has recently been described to achieve a *ZT* equal to 2.96 at 800 K under optimum doping concentration^23^. As seen in the figure, other high-performing thermoelectric materials found in our database are n-type *BiSbSe*_3_ (*ZT* = 2.9 at 750 K)^24^ and *Br*-*doped SnSe* crystals (*ZT* = 2.8 at 773 K)^25^. It is important to note that the *SnSe* variants dominate the discussion for high-performance thermoelecrtic materials, since the publication of a then-unprecedentedly-high thermoelectric figure of merit observed in SnSe single crystals (2.6 at 923 K)^18^ in 2014. Indeed, this is reflected in our database, with more than 76% of the entries with a *ZT* higher than 2.5 involving the *SnSe* compound. The entries with ultra-low thermal conductivity are biased towards false-positives, relative to the rest of the database, due to the power factor of materials sometimes being quoted with units which are standard for thermal conductivity. In terms of magnitude, the power factor is typically much smaller than the thermal conductivity, which creates a bias when examining the end range of low thermal conductivities. Nonetheless, some of the data entries with valid ultra-low thermal conductivities found in our database are: *CuGaTe*_2_ (1.35 Ã— 10^−3^ *Wm*^−1^K^−2^ at 950 K)^26^, *K*_2_*Ti*_6_*O*_13_ whiskers (0.017 *Wm*^−1^ *K*^−2^ at 1033 K)^27^, and *Co*_0.7_*Fe*_0.3_*Sb*_3_ (0.03 *Wm*^−1^ *K*^−2^ at 700 K)^28^.

**Fig. 6:**
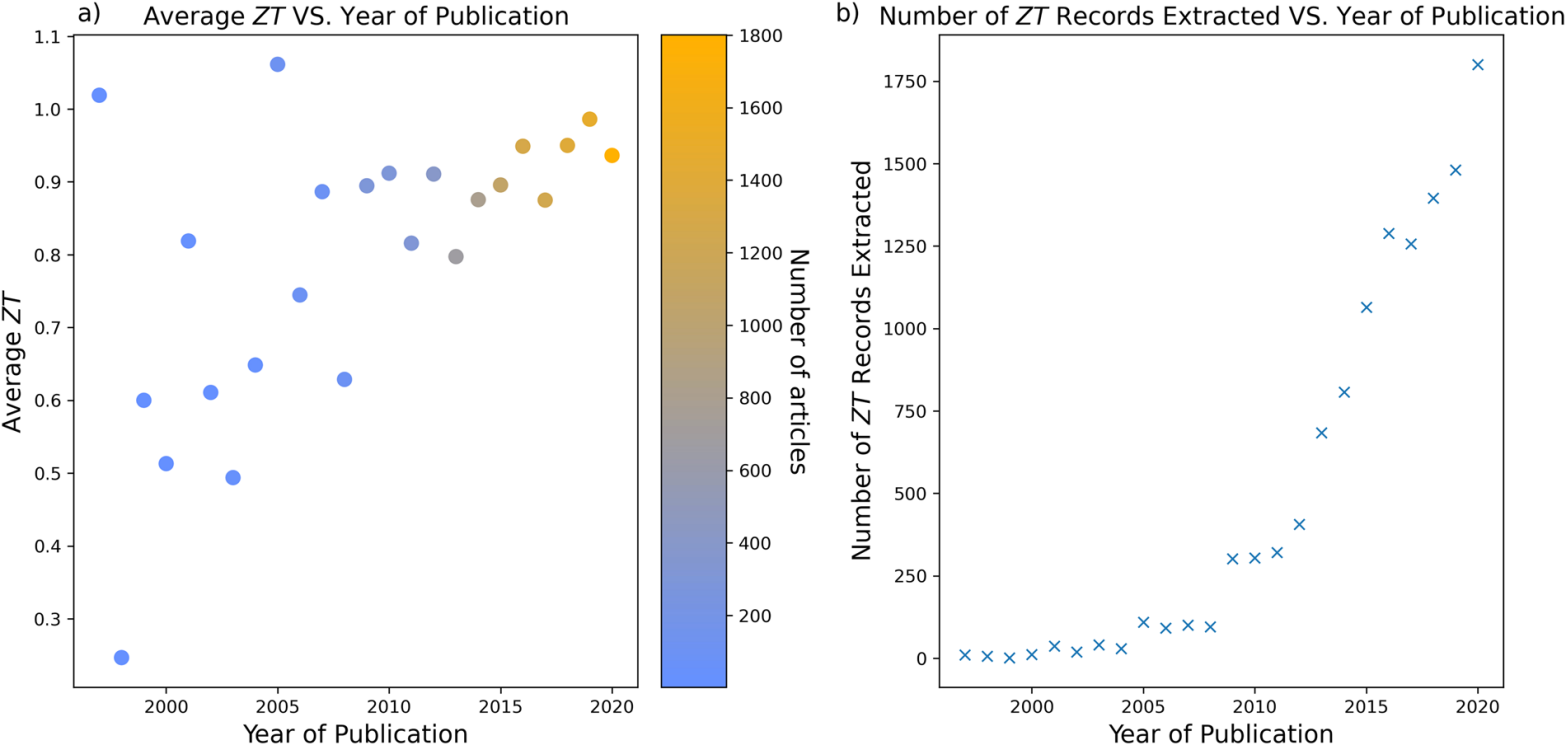
(**a**) Average *ZT* versus year of publication, with marker colours indicating the number of data records per year, according to the colour bar. (**b**) Plot of number of data records per year of publication.

Figure 6 shows the average *ZT* of all materials extracted against their year of publication, between the years 1997 and 2020, (Fig. 6a) as well as the number of records per year (Fig. 6b). Clearly, a positive trend can be identified in *ZT*, which implies that the efforts of the thermoelectric-materials community tend to successful progress. The outliers in the years 1997 and 2005 can be disregarded, since there are very few records per year before 2010, so the averaging process is poor where large standard deviations dominate. Extrapolating a linear fit to the data in Fig. 6 (a) predicts that by 2052, the *average* value of thermoelectric materials discussed in literature will be 1.5. Data science methods which can be facilitated by databases such as ours aim to accelerate this trend, through streamlining materials discovery, towards a more sustainable future.

## Usage Notes

The databases have been made available in CSV, JSON, and MongoDB formats; in both full and aggregated-with-inferences versions, and can be downlaoded from *Figshare*^21^. The full version is the main database, discussed in the Data Records Section. These databases can be queried by data query languages, such as SQL or the MongoDB Query Language; as well as programming languages with the appropriate support, such as Python, R, Java, MATLAB, or others. The data records in the databases are easy to read, search, edit, and delete, thanks to the structured forms of the databases.

## Supplementary information

Supplementary Information

## Data Availability

The code used to automatically generate the database is available at https://github.com/odysie/thermoelectricsdb, along with examples, code for cleaning and aggregating the database, and supplementary information about the database and the data extraction process.

## References

[1] Beretta D (2019). Thermoelectrics: From history, a window to the future. Materials Science and Engineering: R: Reports.

[2] Rowe, D. M. *CRC handbook of thermoelectrics* (CRC press, 2018).

[3] Alam H, Ramakrishna S (2013). A review on the enhancement of figure of merit from bulk to nano-thermoelectric materials. Nano Energy.

[4] Alpaydin, E. *Introduction to machine learning* (MIT press, 2020).

[5] Gaultois MW (2013). Data-driven review of thermoelectric materials: performance and resource considerations. Chemistry of Materials.

[6] Gaultois, M. W. *et al*. A recommendation engine for suggesting unexpected thermoelectric chemistries. *arXiv preprint arXiv:1502.07635* (2015).

[7] Hautier, G. Prediction of new battery materials based on ab initio computations. In *AIP Conference Proceedings*, vol. 1765, 020009 (AIP Publishing LLC, 2016).

[8] Ong SP (2013). Python materials genomics (pymatgen): A robust, open-source python library for materials analysis. Computational Materials Science.

[9] Carrete J, Mingo N, Wang S, Curtarolo S (2014). Nanograined half-heusler semiconductors as advanced thermoelectrics: An ab initio high-throughput statistical study. Advanced Functional Materials.

[10] Gorai P (2016). Te design lab: A virtual laboratory for thermoelectric material design. Computational Materials Science.

[11] Yan J (2015). Material descriptors for predicting thermoelectric performance. Energy & Environmental Science.

[12] Tshitoyan V (2019). Unsupervised word embeddings capture latent knowledge from materials science literature. Nature.

[13] Swain MC, Cole JM (2016). ChemDataDxtractor: a toolkit for automated extraction of chemical information from the scientific literature. Journal of Chemical Information and Modeling.

[14] Mavračić J, Court CJ, Isazawa T, Elliott SR, Cole JM (2021). ChemDataExtractor 2.0: Autopopulated ontologies for materials science. Journal of Chemical Information and Modeling.

[15] Agichtein, E. & Gravano, L. Snowball: Extracting relations from large plain-text collections. In *Proceedings of the fifth ACM conference on Digital libraries*, 85–94 (2000).

[16] Court CJ, Cole JM (2018). Auto-generated materials database of Curie and Néel temperatures via semi-supervised relationship extraction. Scientific Data.

[17] Huang S, Cole JM (2020). A database of battery materials auto-generated using ChemDataExtractor. Scientific Data.

[18] Zhao L-D (2014). Ultralow thermal conductivity and high thermoelectric figure of merit in SnSe crystals. Nature.

[19] From resources of the Argonne Leadership Computing Facility, which is a DOE office of science user facility supported under contract DE-AC02-06CH11357.

[20] Lisandro Dalcin and Mikael Mortensen. mpi4py-fft.

[21] Sierepeklis O, Cole JM (2022). figshare.

[22] Cole JM (2020). A design-to-device pipeline for data-driven materials discovery. Accounts of Chemical Research.

[23] Yang J (2020). Excellent thermoelectric performance of bamgsi driven by low lattice thermal conductivity: A promising thermoelectric material. Journal of Alloys and Compounds.

[24] Zhang Z, Zhang R, Qi N, Wu Y, Chen Z (2020). Microscopic origin of the extremely low thermal conductivity and outstanding thermoelectric performance of BiSbX_3_ (X = S, Se) revealed by first-principles study. Physical Chemistry Chemical Physics.

[25] Chang C (2018). 3d charge and 2d phonon transports leading to high out-of-plane ZT in n-type SnSe crystals. Science.

[26] Plirdpring T (2012). Chalcopyrite CuGaTe2: a high-efficiency bulk thermoelectric material. Advanced Materials.

[27] Li J (2019). Substantial enhancement of mechanical properties for SnSe based composites with potassium titanate whiskers. Journal of Materials Science: Materials in Electronics.

[28] Kim I-H, Ur S-C (2007). Electronic transport properties of Fe-doped CoSb_3_ prepared by encapsulated induction melting. Materials Letters.

