# Supplementary material for: A thermoelectric materials database auto-generated from the scientific literature using ChemDataExtractor
Source: Sci Data. 2022 Oct 22;9:648. doi: 10.1038/s41597-022-01752-1 (PMC9587980; doi:10.1038/s41597-022-01752-1)
Supplement: Supplementary file 1 — Supplementary Information [file 41597_2022_1752_MOESM1_ESM.docx]

Supplementary Material:

<https://github.com/odysie/thermoelectricsdb/tree/main/supplementary_material>
